# Impact of pre‐examination video education in Gd‐EOB‐DTPA‐enhanced liver MRI: A comparative study

**DOI:** 10.1002/jmrs.833

**Published:** 2024-11-11

**Authors:** Hongfang Huang, Chenhui Li, Zisan Zeng, Junli Liang

**Affiliations:** ^1^ Department of Radiology The First Affiliated Hospital of Guangxi Medical University Nanning China

**Keywords:** anxiety, gadoxetate disodium, image quality, liver magnetic resonance imaging, pre‐examination care, satisfaction, video guidance

## Abstract

**Introduction:**

Hepatocellular carcinoma (HCC) is a leading cause of cancer‐related mortality, and early diagnosis via gadolinium ethoxybenzyl‐diethylenetriamine pentaacetic acid (Gd‐EOB‐DTPA)‐enhanced magnetic resonance imaging (MRI) significantly impacts patient outcomes. However, patient anxiety during MRI can affect image quality. This study investigates the impact of pre‐examination video education on anxiety, satisfaction and image quality in Gd‐EOB‐DTPA‐enhanced liver MRI.

**Methods:**

We prospectively enrolled 480 patients who underwent Gd‐EOB‐DTPA‐enhanced liver MRI from January 2022 to May 2023 at our hospital. Patients were divided into study and control groups in order of odd and even days, with 240 cases in each group. Before the examination, the radiology staff provided routine verbal guidance and breathing training to the patients in the control group, while the study group was given additional video education. The state anxiety scores, satisfaction scores of the provided information and motion artefact scores of the images before and after the examination were compared between the two groups.

**Results:**

The state anxiety scores of both groups of patients were lower than before the examination (all *P* < 0.05), but the change value of the study group was significantly greater than that of the control group (*P* = 0.004). The satisfaction rate of the information provided before the scan in the study group was significantly higher (*P* < 0.001). The image quality scores of the arterial phase were similar between the two groups (*P* = 0.403), but the image quality of the study group in the pre‐contrast, portal phase, transitional phase and hepatobiliary phase was significantly better than that of the control group (all *P* < 0.05).

**Conclusion:**

Supplementing routine pre‐scan care with video guidance for Gd‐EOB‐DTPA‐enhanced liver MRI offers several benefits, including reduced patient anxiety, increased satisfaction and improved image quality. These results suggest the potential for widespread application of video‐based interventions to enhance the MRI experience for patients.

## Graphical abstract

This study evaluates the effects of pre‐examination video education on patient anxiety, satisfaction and image quality in Gd‐EOB‐DTPA‐enhanced liver MRI. We found that video education significantly reduced anxiety, increased patient satisfaction and improved image quality compared to standard pre‐MRI guidance, highlighting the potential of video interventions to optimise the MRI experience.

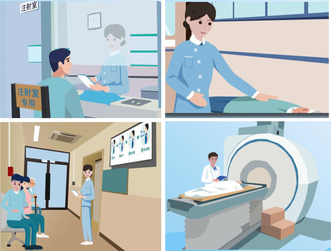

## Introduction

Hepatocellular carcinoma (HCC), the most prevalent primary malignant liver tumour, is the third leading cause of cancer‐related mortality globally.^1^ Early detection and treatment of HCC significantly improve the 5‐year survival rate. According to a meta‐analysis, magnetic resonance imaging (MRI) has a considerably higher sensitivity for the diagnosis of HCC compared to CT. Moreover, gadolinium ethoxybenzyl‐diethylenetriamine pentaacetic acid (Gd‐EOB‐DTPA)‐enhanced MRI showed significantly greater sensitivity than conventional contrast‐enhanced MRI.^2^ Gd‐EOB‐DTPA is a liver‐specific contrast agent that demonstrates selective accumulation within hepatocytes. This selective uptake allows for the enhanced visualisation of certain characteristics of HCC, such as tumour margins and internal structures, which may not be as discernible with non‐specific contrast agents. Consequently, radiologists are better equipped to identify and differentiate HCC from other liver lesions when assessing MRI images. The distinctive features highlighted by Gd‐EOB‐DTPA contribute to its growing preference in diagnostic imaging for HCC.^3^

Despite the proven benefits of Gd‐EOB‐DTPA in enhancing the diagnostic accuracy of liver MRI, the patient experience during MRI scans is a critical factor that can influence the quality of imaging and, consequently, the diagnostic outcome. Anxiety and discomfort associated with the MRI process, including the enclosed space of the scanner, the loud noises produced by the MRI machine, the prolonged duration, restricted mobility and novelty of the environment, are well‐documented challenges that can lead to suboptimal breath‐holding by patients and inferior image quality. These issues are particularly pronounced in the context of Gd‐EOB‐DTPA‐enhanced scans, where the need for precise imaging to capitalise on the contrast agent's liver‐specific properties is paramount. Patient education has been identified as a key intervention to mitigate these anxieties and improve scan outcomes. Moreover, Gd‐EOB‐DTPA has been reported to cause acute transient dyspnoea after intravenous administration, which can affect the arterial phase image quality.^4^ Given that Gd‐EOB‐DTPA is more expensive than conventional contrast agents, the need for repeat scans due to motion artefacts or poor breath‐holding not only wastes valuable time and resources but also increases the cost of the procedure. Therefore, ensuring optimal patient cooperation and minimising discomfort during the scan are critical to fully leveraging the advantages of Gd‐EOB‐DTPA‐enhanced liver MRI and to avoid the economic and diagnostic implications of substandard imaging.

Patient education has been identified as a key intervention to mitigate these anxieties and improve scan outcomes. Radiology staff, including radiographers and nurses, engage in patient communication before scanning, educating them about the clinical intervention process and purpose. This approach aims to mitigate anticipated anxiety, enhance patient enthusiasm and improve cooperation during MRI scans, thus improving overall satisfaction.^5^ In addition, a study has shown that the use of breathing training before Gd‐EOB‐DTPA‐enhanced liver MRI examination can significantly reduce respiratory motion artefacts.^6^ However, the pressure to enhance efficiency and patient flow in most healthcare facilities often leads to limited time for communication between radiology staff and patients before examinations, resulting in insufficiently detailed guidance and training for patients. To address these issues, cost‐effective video interventions have been integrated into the preparatory process before magnetic resonance imaging. Moreover, video education has been identified as a key intervention to mitigate these anxieties and improve scan outcomes.^7^, ^8^, ^9^, ^10^

While other studies have explored the use of video or visual interventions to reduce anxiety and improve imaging in MRI, they have not focused on the unique challenges posed by Gd‐EOB‐DTPA. In light of these considerations, our study specifically targets Gd‐EOB‐DTPA‐based liver MRI scans to investigate the impact of pre‐examination video education on patient anxiety, satisfaction and image quality. By addressing these specific concerns, our study aims to contribute to the body of literature by demonstrating the potential benefits of targeted video education in enhancing the MRI experience for patients undergoing Gd‐EOB‐DTPA‐enhanced liver scans.

## Materials and Methods

### Participants

The study enrolled both inpatients and outpatients who underwent Gd‐EOB‐DTPA‐enhanced liver MRI examination between January 2022 and May 2023. Inclusion criteria comprised patients with no prior experience of liver magnetic resonance imaging, as previous exposure might influence anxiety levels and breath‐holding capacity. Additionally, eligible participants were aged over 18 years, proficient in Chinese language and capable of independently completing four survey questionnaires. Exclusion criteria encompassed patients with significant ascites that could impede breath‐holding ability and those with contraindications to the examination. To ensure impartiality, patients receiving magnetic resonance imaging on odd days were assigned to the control group, while those on even days were allocated to the study group, thereby preventing any interactions between subjects in the two groups. The final sample size comprised 240 patients in each group, summing up to 480 participants for the study.

### Procedure

#### Registration desk

Patients are instructed to arrive at the MRI registration desk 60 min prior to their scheduled examination time, where they complete the Trait Anxiety Inventory (TAI), State Anxiety Inventory prior to the examination (SAI‐pre) and a comprehensive survey questionnaire. The Trait Anxiety Inventory, encompassing 20 questions (see Appendix S1), gauges the respondent's propensity for anxiety, with responses indicating the frequency of ‘in general’ feelings, ranging from ‘none at all’ to ‘always’ (scores 1–4). The score range for this inventory is 20–80, with higher scores indicating heightened anxiety susceptibility. The State Anxiety Inventory, designed to assess anxiety experienced at a particular moment, measures the respondent's ‘current’ feelings, graded on a scale from ‘none at all’ to ‘very obvious’ (scores 1–4), and yields a potential score range of 20 to 80. In this experiment, the State Anxiety Inventory was employed to measure changes in patient anxiety levels from their arrival at the radiology department until the conclusion of the scan. Additionally, the survey questionnaire collected information pertaining to the patient's gender, age, educational level, presence of an accompanying individual and the perceived purpose of the examination, classified into two options: (I) uncertainty about liver health status, considering the examination as a means of diagnosing normal liver conditions, and (II) awareness of liver disease, perceiving the examination as a diagnostic measure to assess disease severity. The entire process is designed to take no more than 15 min.

#### Nursing station

Following routine care, including vital sign measurements and intravenous access, at the nursing station, the radiology staff administered a standardised verbal informed consent procedure. The procedure entailed providing the patient with essential information, including (1) MRI examination's non‐ionising radiation nature, (2) the need to remove all metal objects before entering the scanning room, with a safe space provided, (3) the experience of entering the scanner, akin to a tunnel but non‐hazardous, with an experienced technician monitoring from the control panel, (4) the typical occurrence of loud noises similar to knocking during the scan, which are normal scanner functions and (5) the requirement to lie in the scanner for approximately 30 min while cooperating with the machine's breathing instructions to obtain high‐quality images. Subsequently, the nurse spent 1–2 min guiding and supervising the patient to complete at least one full breathing exercise to optimise the examination experience. This nursing station procedure, including the informed consent and breathing exercise, is intended to be completed within a maximum of 15 min.

#### Independent waiting room

Following the nursing station procedures, patients entered an independent waiting room to await the scan. In the case of the study group, patients watched a video displayed on a TV screen, with a duration of approximately 20 min and split into two segments. The first segment intuitively demonstrated the MRI machine's appearance, working principle and provided examples of machine noises, along with patient requirements during the scan. The second segment showcased the machine's breathing instructions during the scan and illustrated the impact of inadequate breath‐holding on image quality and diagnosis, followed by actor demonstrations of effective breathing training methods for patients. Conversely, patients in the control group did not view the video during their waiting period. Both groups rested in the waiting area for a minimum of 30 min before proceeding to the MRI room. After a 30‐min wait in the waiting room, all patients were asked to complete the second State Anxiety Inventory (SAI‐post) using the same evaluation items as before.

#### MRI room

After necessary preparation, patients entered the MRI room, where the MRI machine, Magnetom Prisma 3.0 T (Siemens Medical, 2018), was utilised for the scan. MRI technicians with over a decade of experience in abdominal scanning conducted the scan following standard sequences. In cases where patients failed to comply with the breathing instructions, leading to severe motion artefacts in the images, technicians repeated the scan for optimal image quality.

#### Observation area

Following completion of the scan, patients proceeded to the observation area, where they remained for a minimum of 20 min. Once the nurse confirmed the absence of any physical discomfort arising from the scan, patients were allowed to leave. Prior to departure, all patients were required to complete the second State Anxiety Inventory (SAI‐post) and a satisfaction questionnaire, featuring identical evaluation items. The satisfaction questionnaire assessed patients’ contentment with the information provided by the nurse before the scan and the video. It consisted of three closed questions, each with three ordered response categories scored 1–3. The total score of the three questions ranged from 3 to 5 points for dissatisfaction, 6 to 8 points for satisfaction and 9 points for very satisfaction.

### Image quality assessment

**Figure 1:**
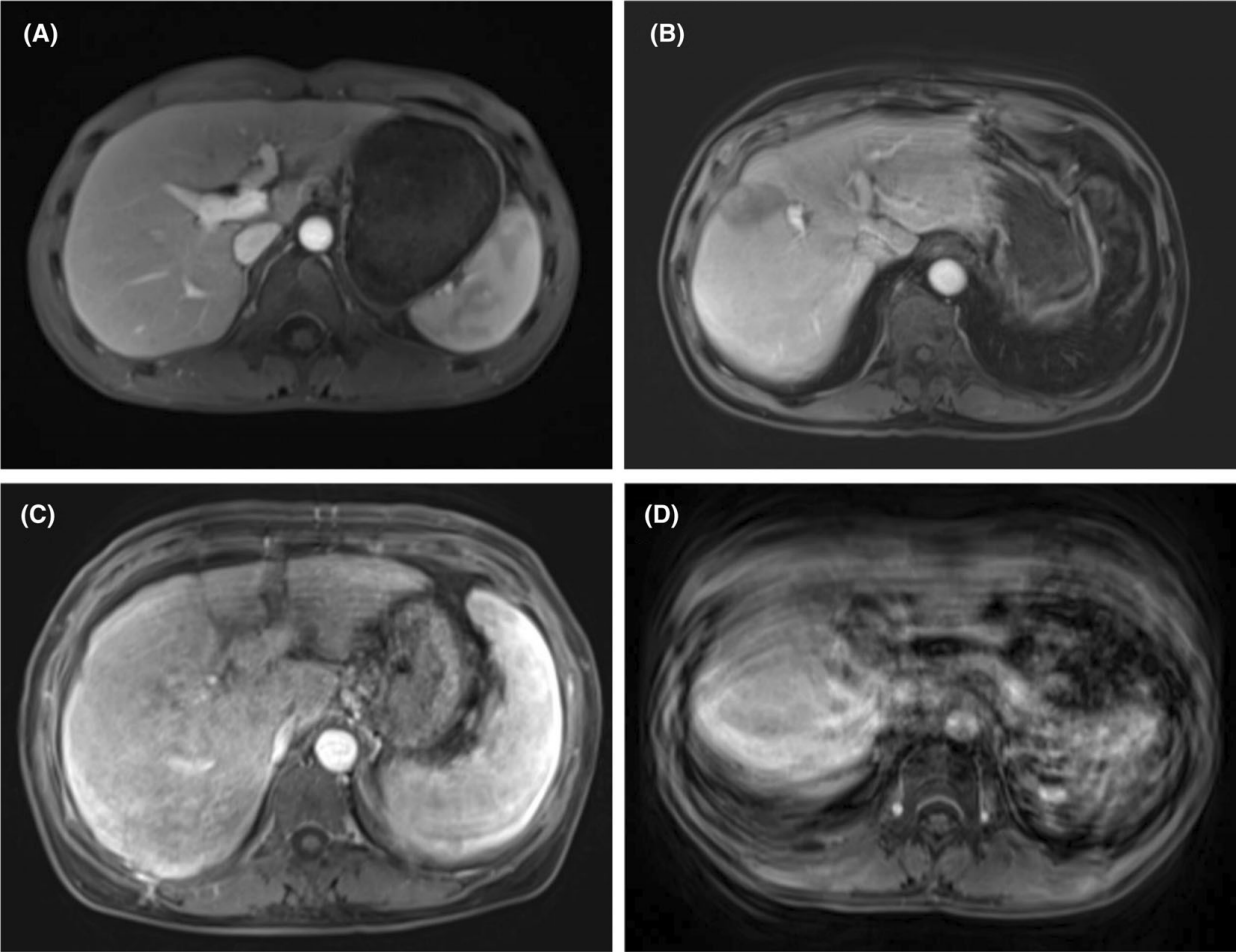
Motion artefact scoring criteria. 1 point = no motion artefacts (A); 2 points = mild motion artefacts with no impact on diagnosis (B); 3 points = moderate motion artefacts with some impact on diagnostic quality but not severe (C); and 4 points = severe motion artefacts with the image being non‐diagnostic (D).

Two specialist abdominal radiologists with over 5 years of experience, who were blind to the group as well as the intervention that the patient received, independently assessed the image quality of the dynamic T1‐weighted sequence, which encompassed the pre‐contrast, arterial phase, portal phase, transitional phase and hepatobiliary phase. A specific scoring system was employed for this assessment (see Fig. 1 for details). Each imaging sequence's motion artefact score was then averaged between the two observers, ensuring a comprehensive and objective evaluation of image quality.

### Ethical considerations

The present study received ethical approval from the Ethics Committee of the First Affiliated Hospital of Guangxi Medical University and adhered to the tenets delineated in the Helsinki Declaration (World Medical Association, 2001). Prior to participation, all enrolled patients received a comprehensive elucidation of the study procedures, and written informed consent was obtained from each individual. Participants were apprised of the voluntary nature of their involvement and were assured that they retained the prerogative to withdraw from the study at any juncture without incurring any adverse repercussions.

### Data analysis

Data analysis was performed using SPSS 23.0. Categorical data were presented as case numbers and percentages (%) and were subjected to chi‐square tests for analysis. Metric data were expressed as mean ± standard deviation (*x* ± *s*) and were analysed using either *t*‐tests or Mann–Whitney *U* tests. A significance level of *P* < 0.05 was considered indicative of statistically significant differences. To assess the inter‐rater reliability of motion artefact scores among different radiologists, the intra‐class correlation coefficient (ICC) was employed, where ICC values falling within the range of 0–0.4 denoted poor consistency, 0.4–0.75 indicated general consistency, and values exceeding 0.75 suggested very good consistency.^11^

## Results

Table 1 reveals a lack of discernible differences between the two patient groups concerning gender, age, educational level, accompanying personnel, examination purpose and trait anxiety.

**Table 1:** Patient characteristics and scan time in the study and control groups.

	Study group *n* (%) (*n* = 240)	Control group *n* (%) (*n* = 240)	*P*
pSex			
Male	184 (76.7)	173 (72.1)	0.250^a^
Female	56 (23.3)	67 (27.9)	
Age (years)	45.20±10.77	46.08±11.90	0.671^b^
Education			
Lower than high school	80 (33.3)	96 (40.0)	0.119^a^
High school	76 (31.7)	80 (33.3)	
Higher than high school	84 (35.0)	64 (26.7)	
Presence of relatives			
Yes	81 (33.8)	76 (31.7)	0.627^a^
No	159 (66.2)	164 (68.3)	
Purpose of the examination			
I	18 (7.5)	12 (5.0)	0.258^a^
II	222 (92.5)	228 (95.0)	
TAI scores	54.42±11.98	55.56±13.89	0.629^b^

^a^ Chi‐squared test.

^b^ Independent sample *t*‐test.

Table 2 presents the SAI scores of patients before and after nursing intervention. Notably, there were no significant disparities in SAI‐pre scores between the study group and the control group (57.50 ± 9.08 vs. 58.10 ± 9.71, *P* = 0.729). However, the SAI‐post scores of the study group were significantly lower than those of the control group (45.70 ± 9.32 vs. 53.60 ± 9.17, *P* < 0.001). Moreover, when scrutinising the changes in SAI‐pre and SAI‐post, it becomes evident that both groups experienced decreased SAI‐post scores compared to SAI‐pre scores, with the study group demonstrating a considerably larger reduction than the control group (−11.80 ± 13.59 vs. −4.50 ± 13.72, *P* = 0.004).

**Table 2:** SAI results.

	Study group (*n* = 240)		Control group (*n* = 240)		*P* value (between group)
	Mean ± SD	*P* value (within group)	Mean ± SD	*P* value (within group)	
SAI‐pre	57.50 ± 9.08	–	58.10 ± 9.71	–	0.729^a^
SAI‐post	45.70 ± 9.32	<0.001^b^	53.60 ± 9.17	0.014^b^	<0.001^a^
Chang in SAI	−11.80 ± 13.59	–	−4.50 ± 13.72	–	0.004^a^

SAI‐pre: State Anxiety Inventory when arriving at the registration office room. SAI‐post: State Anxiety Inventory at least 20 min after entering the waiting room.

^a^ Independent sample *t*‐test, between study and control.

^b^ Paired sample *t*‐tests, between SAI‐pre and SAI‐post (both for study and control).

Table 3 showcases the outcomes of the satisfaction survey, indicating that 88.8% of participants in the study group and 71.7% in the control group reported feeling satisfied or very satisfied with the preparation and information provided before the scan. The overall satisfaction rate was markedly higher in the study group than the control group (*P* < 0.001).

**Table 3:** Patient satisfaction.

	Study group *n* (%) (*n* = 240)	Control group *n* (%) (*n* = 240)	*χ* ^2^	*P*
Very satisfied	82 (34.1)	46 (19.2)	–	–
Satisfied	131 (54.6)	126 (52.5)	–	–
Dissatisfied	27 (11.3)	68 (28.3)	–	–
Overall satisfaction	213 (88.8)	172 (71.7)	22.061	<0.001

**Figure 2:**
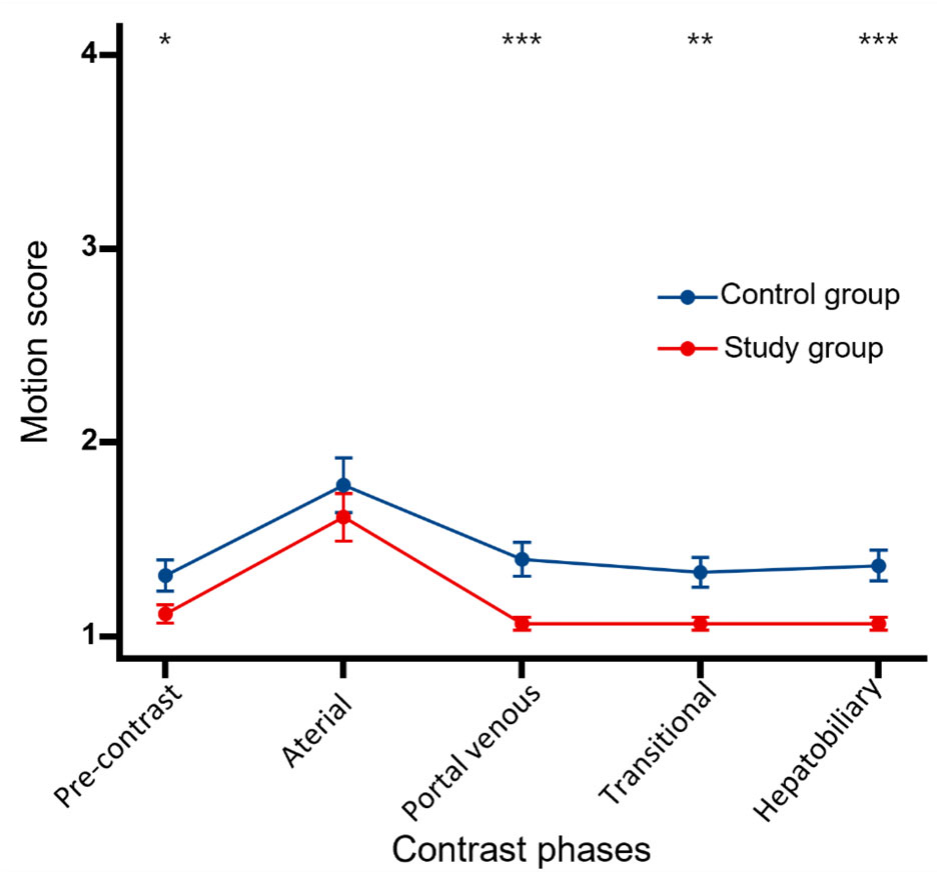
Mean motion artefact scores of study and control groups. *P < 0.05; **P < 0.005; and ***P < 0.001.

The summary of motion artefact scores is displayed in Table 4 and Figure 2. The inter‐observer agreement for motion artefact scores demonstrated excellence across all phases of dynamic enhancement scans. In particular, motion artefact scores in the study group were lower than those in the control group during pre‐contrast, portal phase, transitional phase and hepatobiliary phase, with statistically significant differences (all *P* < 0.05). The arterial phase exhibited the highest motion artefact scores in both the study and control groups, with no significant difference detected between the two groups in this phase (study group: 1.62 ± 0.96 vs. control group: 1.78 ± 1.09; *P* = 0.403).

**Table 4:** Mean motion scores.

	Study group (*n* = 240)		Control group (*n* = 240)		*P* value
	Mean motion score	ICC	Mean motion score	ICC	
Pre‐contrast	1.12 ± 0.37	0.917	1.32 ± 0.62	0.824	0.044^a^
Arterial	1.62 ± 0.96	0.797	1.78 ± 1.09	0.788	0.403^a^
Portal venous	1.07 ± 0.25	0.906	1.40 ± 0.67	0.869	<0.001^a^
Transitional	1.07 ± 0.26	0.864	1.33 ± 0.60	0.913	0.003^a^
Hepatobiliary	1.08 ± 0.25	0.948	1.37 ± 0.61	0.922	<0.001^a^

^a^ Mann–Whitney *U* test.

## Discussion

Anxiety is a common concern among patients undergoing MRI examinations, and previous studies have highlighted the association between anxiety and the provision of information.^12^ Our findings support the notion that supplying patients with scan‐related information by nursing staff prior to the examination can effectively alleviate patient anxiety. Notably, both patient groups exhibited significantly reduced SAI scores after the scan. However, our study also revealed that additional video guidance intervention resulted in a further reduction in patient anxiety, consistent with findings from other relevant literature.^13^ Unlike regular verbal guidance, which may vary in its impact depending on the communication abilities of medical staff and patient understanding, videos with vivid and concrete imagery can evoke greater interest and enthusiasm in patients, promoting effective learning.

The higher satisfaction scores observed in the study group, as compared to the control group, suggest that patients who watched the video guidance before the scan gained a better understanding of the MRI process and had more accurate expectations, leading to reduced anxiety during the examination. Nevertheless, it is essential to emphasise that video guidance cannot replace the need for good communication between radiology staff and patients.^5^

Interestingly, our study found that the study group patients exhibited lower motion artefact scores in their scan images. However, it is important to note that this does not imply a direct causal relationship between anxiety and motion artefacts, as such a connection has not been consistently observed in other studies.^7^, ^14^ Our study results align with the work of Tornqvist et al.,^15^ which showed that providing additional written information to patients before MRI reduced motion artefacts but did not result in reduced anxiety or improved satisfaction. Similarly, the study by Yakar et al.^9^ suggested that visual information might be more effective than written information in reducing MRI anxiety. Therefore, in our study, we developed a more intuitive and easily comprehensible video content compared to written information. The first part of the video aimed to familiarise patients with the scan room environment, thus alleviating anxiety related to the unknown situation. The second part focused on explaining the causes of motion artefacts, their impact on diagnosis and techniques to avoid them, thus assisting patients in better complying with breathing instructions during the scan. As expected, patients who watched the video experienced lower anxiety levels, higher satisfaction and fewer motion artefacts than those in the control group. Notably, our study highlighted that motion artefact scores in arterial phase images were not improved. This phenomenon might be associated with gadoxetate disodium, leading to patients experiencing Transient Severe Respiratory Motion (TSM) during the arterial phase.^16^ Nevertheless, the underlying cause of TSM remains uncertain, and previous research has shown that educational information and breath‐holding training cannot alleviate this issue,^17^ which is consistent with our study findings.

Despite the valuable insights gained from our study, certain limitations warrant consideration. The timing and location of video provision to patients may play a crucial role. In our study, patients watched the video before the examination, resulting in extended waiting times. Additionally, arranging patients to watch the video in an independent waiting room might not be feasible in all hospitals, particularly those lacking such facilities, making it challenging for patients to concentrate amidst a noisy waiting hall. Therefore, we recommend providing patients with a network link to the video during appointment scheduling. This approach not only affords patients sufficient preparation time for viewing the video but also allows for the availability of different language versions, catering to a broader patient population and facilitating dissemination across multiple medical institutions. Further investigations in this regard will be pursued to enhance our understanding of this aspect.

## Conclusion

This study demonstrates the positive impact of providing supplementary video guidance to patients undergoing routine pre‐examination care before Gd‐EOB‐DTPA‐enhanced MRI of the liver. The video intervention effectively reduced patient anxiety, increased satisfaction with the provided information and improved image quality in specific phases of the MRI. Video guidance serves as a valuable and cost‐effective strategy to enhance the overall patient experience and optimise clinical outcomes during Gd‐EOB‐DTPA‐enhanced MRI examinations. Further exploration of the video's role in enhancing patient‐centred care is warranted, and its potential incorporation into routine clinical practice merits consideration.

## Funding Statement

This work was supported by the Self‐funded Scientific Research Section of Guangxi Zhuang Autonomous Region Health Committee (No. Z20190411) and the Open Project of NHC Key Laboratory of Thalassemia Medicine (GJWJWDP202307).

## Conflict of Interest

The authors declare no conflict of interest.

## Ethics Approval and Consent to Participate

This study was approved by the Ethics Committee of the First Affiliated Hospital of Guangxi Medical University (No. 2023‐E407‐01).

## Human and Animal Rights

This study was conducted in accordance with the Declaration of Helsinki principles, and informed consent was obtained from all participating individuals.

## Supporting information


**Appendix S1.** Survey questionnaire – trait anxiety inventory (TAI).

## Data Availability

The data that support the findings of this study are available from the corresponding author upon reasonable request.
