# Supplementary material for: Impact of pre‐examination video education in Gd‐EOB‐DTPA‐enhanced liver MRI: A comparative study
Source: J Med Radiat Sci. 2024 Nov 11;72(1):34–41. doi: 10.1002/jmrs.833 (PMC11909707; doi:10.1002/jmrs.833)
Supplement: Supplementary file 1 — Appendix S1. Survey questionnaire – trait anxiety inventory (TAI). [file JMRS-72-34-s001.docx]

**Survey Questionnaire - TAI (Trait)**

Name: Gender: Male□ Female□ Age: years

Date of Completion: _______________(Year/Month/Day)

Educational Level: Elementary School □ Junior High School □

High School □ University or above □

Have you ever had an experience of undergoing an abdominal magnetic resonance imaging (MRI) examination before this test? Yes □ No □

Instructions: The following are statements that people often use to describe themselves. Please read each statement and select the option that best represents how you feel most of the time. There are no right or wrong answers. Do not spend too much time considering any one statement, but your responses should reflect your usual feelings.

|  | **Never** | **Somewhat** | **Often** | **Always** |
| --- | --- | --- | --- | --- |
| *1. I feel cheerful | 1 | 2 | 3 | 4 |
| 2. I feel nervous and uneasy | 1 | 2 | 3 | 4 |
| *3. I feel self-satisfied | 1 | 2 | 3 | 4 |
| *4. I wish I could be as happy as others | 1 | 2 | 3 | 4 |
| 5. I feel as if I'm exhausted | 1 | 2 | 3 | 4 |
| *6. I feel very calm | 1 | 2 | 3 | 4 |
| *7. I am quiet, composed, and at ease | 1 | 2 | 3 | 4 |
| 8. I feel overwhelmed by difficulties | 1 | 2 | 3 | 4 |
| 9. I worry excessively about unimportant matters | 1 | 2 | 3 | 4 |
| *10. I am happy | 1 | 2 | 3 | 4 |
| 11. My mind is in a state of confusion | 1 | 2 | 3 | 4 |
| 12. I lack self-confidence | 1 | 2 | 3 | 4 |
| *13. I feel secure | 1 | 2 | 3 | 4 |
| *14. I make decisions easily | 1 | 2 | 3 | 4 |
| 15. I feel out of place | 1 | 2 | 3 | 4 |
| *16. I am content | 1 | 2 | 3 | 4 |
| 17. Unimportant thoughts constantly occupy my mind and disturb me | 1 | 2 | 3 | 4 |
| 18. The depression I feel is so intense that I cannot get it out of my mind | 1 | 2 | 3 | 4 |
| *19. I am a calm person | 1 | 2 | 3 | 4 |
| 20. When I think about my current affairs and interests, I become tense | 1 | 2 | 3 | 4 |
